# Facilitators and barriers in anorexia nervosa treatment initiation: a qualitative study on the perspectives of patients, carers and professionals

**DOI:** 10.1186/s40337-021-00381-0

**Published:** 2021-02-27

**Authors:** Denise Kästner, Angelika Weigel, Ines Buchholz, Ulrich Voderholzer, Bernd Löwe, Antje Gumz

**Affiliations:** 1 Department of Psychosomatic Medicine and Psychotherapy, University Medical Centre, Hamburg-Eppendorf, Martinistr. 52, W37, 20246 Hamburg, Germany; 2 Schön Clinic Roseneck, Prien am Chiemsee, Germany; 3 Department of Psychiatry and Psychotherapy, University Hospital Freiburg, Freiburg, Germany; 4 Department of Psychiatry and Psychotherapy, University of Munich (LMU), Munich, Germany

**Keywords:** Anorexia nervosa, Early intervention, Facilitators and barriers, Psychotherapy, Qualitative study

## Abstract

**Background:**

An early psychotherapeutic treatment of anorexia nervosa (AN) is crucial for a good prognosis. In order to improve treatment initiation, knowledge about facilitators and barriers to treatment is needed.

**Objective:**

Against this background, we aimed to identify facilitators and barriers from the perspectives of patients, carers and professionals using a qualitative approach.

**Method:**

To this end, semi-structured interviews were conducted in triads of female patients with AN aged 14 years and older at the beginning of their first psychotherapeutic treatment, their carers, and referring health care professionals. A modified Grounded Theory approach was used for analysis.

**Results:**

In total, 22 interviews were conducted (*n* = 6 adults, *n* = 4 adolescents, 4 full triads). The duration of untreated AN ranged between 30 days and 25.85 years (M = 3.06 ± 8.01 years). A wide spectrum of facilitators and barriers within the patient, the social environment, the health care system and the society were identified. Most prominent factors were ‘recognizing and addressing’ by close others, ‘waiting times and availability’ and ‘recommendations and referrals’ by health care professionals. ‘Positive role models for treatment’ were perceived as a specific facilitative social influence. Facilitators were more frequently mentioned than barriers and most of the factors seem to hold potential for modifiability.

**Conclusion:**

Overall, the findings suggest that early intervention approaches for AN should not only address patients and the health care system, but may also involve carers and successfully treated former patients.

**Trial registration:**

ClinicalTrials.gov Identifier: NCT03713541.

**Supplementary Information:**

The online version contains supplementary material available at 10.1186/s40337-021-00381-0.

## Plain English summary

An early treatment of Anorexia nervosa (AN) can improve the prognosis and avoid a chronic and severe course of illness. In order to decrease the duration of untreated AN, solid knowledge about the existing facilitators and barriers to treatment is essential. This study aimed to identify facilitators and barriers to psychotherapeutic treatment for AN from different perspectives. To this end, we conducted and analyzed *n* = 22 interviews with (*n* = 4) adolescent and (*n* = 6) adult patients at the beginning of their first treatment, their carers (*n* = 7), and referring health care professionals (n = 4). Our findings confirm previous study results on the importance of the social environment during the process of treatment initiation and on relevant barriers within the health care system like long waiting times and negative previous experiences with health care professionals. Facilitating influences not previously considered resulted from successfully treated former patients acting as ‘positive role models for treatment’. If future studies support our findings, successfully treated former patients and carers may be involved more strongly in early intervention approaches for AN.

## Introduction

Anorexia nervosa (AN) is a serious illness characterized by self-induced underweight, body image distortions and fear of weight gain [1]. The long-term outcome of AN is highly variable, but it was estimated that about 20% of all individuals with AN remain chronically ill [2, 3]. Moreover, the mortality rate of AN is higher than in any other mental disorder [4, 5]. The prognosis of patients with AN depends highly on how early specialized AN-treatment starts [6, 7]. Over time, eating disorder pathology can become a learned self-perpetuating rewarding process [8] and various medical complications may emerge [9]. In order to avoid hospital admissions, poor treatment outcomes and chronic courses, it is crucial to treat AN at early illness stages [6, 7].

Compared to other mental disorders (especially schizophrenia), research on early diagnosis and treatment in AN is still relatively scarce [10, 11]. Existing evidence suggests that the first 3 years of AN are the critical time window for intervention [12]. However, the mean duration of untreated AN (DUAN) was found to be approximately two to 3 years [13–16]. The DUAN range is usually wide and some patients do receive their first AN treatment only decades after the illness onset. For a closer look, the DUAN can be further divided into 1) the time span without health care contacts and 2) the time from the first AN-related health care contact to the beginning of the specialized treatment. The first interval was found to be about one to two and a half years, the second interval 10 months to 1 year [13, 17, 18]. Consequently, both close others [15] and general practitioners [17] play a decisive role in early treatment initiation. However, studies investigating their perspectives on the process of treatment initiation are missing [19, 20].

So far, widespread early intervention approaches for patients with AN have yet to be developed and implemented [11]. A single novel service for young adults with eating disorders named “First Episode and Rapid Early Intervention for Eating Disorders” showed highly promising results with respect to a reduction of the DUAN (on average 4 months shorter compared to treatment as usual), reduced treatment disengagement and improved patient satisfaction [21–23]. In order to inform the development and the improvement of such services and other approaches to early diagnosis and treatment, knowledge about existing facilitators and barriers to treatment is crucial. Two recent reviews summarized the current evidence on this topic. Accordingly, feelings of shame and stigma, personal beliefs related to the eating disorder (e.g. failure to perceive the severity of the illness), problems associated with the health care system (lack of availability, cost of treatment), and the lack of encouragement from others were identified as important barriers to treatment. Health-related concerns, other mental health problems and/or emotional distress, and social support were relevant facilitators. However, several weaknesses of the current evidence base were outlined. Firstly, most studies did not focus on specific eating disorders but rather aggregate samples of patients with various diagnoses [20]. Secondly, most studies on facilitators and barriers did not include adolescent patients and thereby potentially ignore age-group specific factors [19]. Finally, the perspectives of professionals or close others were not considered in any study despite their important role in the process of treatment initiation [19, 20].

Against this background, we aim to identify facilitators and barriers to specialized AN treatment from the perspective of adolescent and adult patients, carers and referring professionals. Knowledge about the spectrum of facilitators and barriers from multiple perspectives is crucial for improvements. Furthermore, as the first part of a mixed-methods study (Facilitators And Barriers In Anorexia NervosA treatment initiation - FABIANA-study) the present results will also directly contribute to the development of an instrument to assess facilitators and barriers (FABIANA sub-study II) and subsequently test their influence on the DUAN quantitatively (FABIANA sub-study III). In the long-term, the findings of the FABIANA-study will help to inform early intervention approaches and avoid chronic courses of AN.

## Method

### Study design

The FABIANA-study is a mixed-methods multi-center study with three consecutive sub-studies. The present manuscript reports sub-study I. A detailed overall description can be found within the study protocol [24]. In brief, sub-study I is a qualitative multi-informant study exploring the perspectives of triads of female patients with AN, their carers and referring professionals on facilitators and barriers to treatment initiation using semi-structured interviews. The qualitative approach is useful, common and effective for obtaining rich data on opinions, attitudes, motives, behaviors, and expectations. As part of a mixed-methods design it is a very common first step to develop and inform the quantitative measurement of complex constructs [25]. Data collection and analysis were conducted in line with the Consolidated criteria for reporting qualitative research (COREQ) criteria [26]. Ethical approval was obtained prior to recruitment (PV5108). This manuscript was prepared using the Standards for Reporting Qualitative Research (SRQR) [27].

### Participants

Recruitment involved five cooperating partners who offer specialized psychotherapeutic AN treatment (four inpatient clinics, one psychotherapy training center).

Eligible for study participation were female patients with AN aged 14 years and older, who were at the beginning of their first psychotherapeutic AN treatment (i.e., a) start no longer than 3 months ago; b) at least 7 days of inpatient care or 5 sessions outpatient care completed; c) since illness onset no AN treatment). The eligibility criteria were chosen to guarantee a sufficiently consolidated development of cognitive functions such as abstract thinking and reasoning (minimum age), to minimize the risk of recall biases and response shifts (beginning of the first treatment no longer than 3 months ago) and to exclude early treatment dropouts (minimum number of completed inpatient days or outpatient sessions). AN diagnosis was validated in the context of SCID-interviews. Study participants were non-randomly selected using mixed purposeful sampling. In order to select adolescent and adult AN patients and to achieve variance with respect to the DUAN (< vs. ≥ 1 year) stratified purposeful sampling was used; their carers and professionals were included in the study by network (snowball) sampling [28]. The aim was to include complete triads. Recruitment was terminated with theoretical saturation (i.e. new material could be fully described with existing categories).

### Data collection

All participants and, in case of minor participants, legal guardians gave written informed consent. To assess basic sample characteristics, participants filled out a short questionnaire prior to the interview. The treating psychotherapist provided information on patients’ weight, height and the date of treatment initiation.

Qualitative interviews were based on semi-structured guidelines designed for each perspective. The guidelines were developed by the research team. They were tested in a group consisting of physicians and psychologists and refined accordingly. Interviews were conducted by two female PhD-level psychologists with patients being interviewed face-to-face, and carers and professionals by phone. Interviewers and study participants did not know each other prior to undertaking the study. Prior to data collection, the study team reflected on and noted any a priori assumptions on emerging topics.

Patient interviews were divided into two parts. The first part sought to check inclusion and exclusion criteria and to retrospectively determine the month of AN onset. The procedure is described in detail within the study protocol [24]. The second part of the patient interviews and the interviews with the carers and professionals were concerned with facilitators and barriers for the treatment initiation. At first, all participants were asked to describe the process from the beginning of the first symptoms to the beginning of the treatment with their own words. Afterwards, the interviews focused on factors facilitating or hindering treatment initiation (e.g. “What made it easier/harder for you/your daughter/your friend/your patient to start treatment?”) and became increasingly more specific. All interviews were audio-taped and transcribed using pre-specified transcription rules [29].

### Data analysis

In line with the applied Grounded Theory approach [30, 31], data analysis was carried out simultaneously to the data collection. By that, the already analyzed material influenced the subsequent data collection (e.g. specifically recruit cases that may provide so far missing information). Each interview was analyzed independently by two members of the study team using MAXQDA (software package for Qualitative Data Analysis named after the sociologist MAX Weber) [32]. Analysis started with open coding of the interviews. Memos were written for each interview and as needed for specific codes. Subsequently, the code tree was gradually developed and repeatedly refined in order to reach the best possible fit with the interview material. In total, four female researchers developed and discussed the categories and coded the interviews (three PhD-level psychologists and one professor for psychosomatic medicine and psychotherapy). In order to measure the influence of specific, well-defined facilitators and barriers in the following FABIANA-sub-studies, rather broad aggregations were avoided within the qualitative analysis.

Subsequently, the developed category system (1. main categories, 2. factors, i.e. F = facilitators and B = barriers sorted by the frequency they were mentioned) is presented. Code names (i.e. categories) are indicated by single quotation marks, exemplary quotes by double quotation marks. Exemplary quotes are selected to further illustrate less self-explanatory factors and to substantiate the findings [27]. Quotes are identified by perspective and a study code linking the triad (P = patient, C = carer, Pr = professional). They are edited for legibility and explanations are added in square brackets were necessary.

## Results

### Sample characteristics

In total, 22 interviews were conducted with 10 patients, 7 carers, 4 professionals and one study participant, who was both carer (of an adolescent patient) and professional (4 complete triads: 3 with adult patients, 1 with an adolescent patient). The interviews took on average 1 h and 2 min (SD = 14:33 min).

Among the patients were six adults (mean age = 23.33 years, SD = 7.47, range: 20–38 years; mean BMI = 15.9 kg/m^2^, SD = 2.25) and four adolescents (mean age = 15.75 years, SD = 1.50, range: 14–17 years; mean BMI = 15.6 kg/m^2^, SD = 1.43; mean % expected body weight = 73.41, SD = 4.47). One of the adolescent patients and three of the adult patients had at least one comorbid SCID-I diagnosis. No participant was diagnosed with a personality disorder. Mean DUAN within the sample was 3.06 years (SD = 8.01; median = 0.58 years) with a range between 30 days and 25.85 years. The interviewed carers were four mothers, two fathers and one (female) friend (mean age = 52.00 years, SD = 3.79). The professionals were four general practitioners and one psychotherapist (*n* = 4 female, *n* = 1 male; mean age = 53.40 years, SD = 7.02). Two of the general practitioners were specialized in the treatment of eating disorders.

### Overview of the qualitative results

**Fig. 1:**
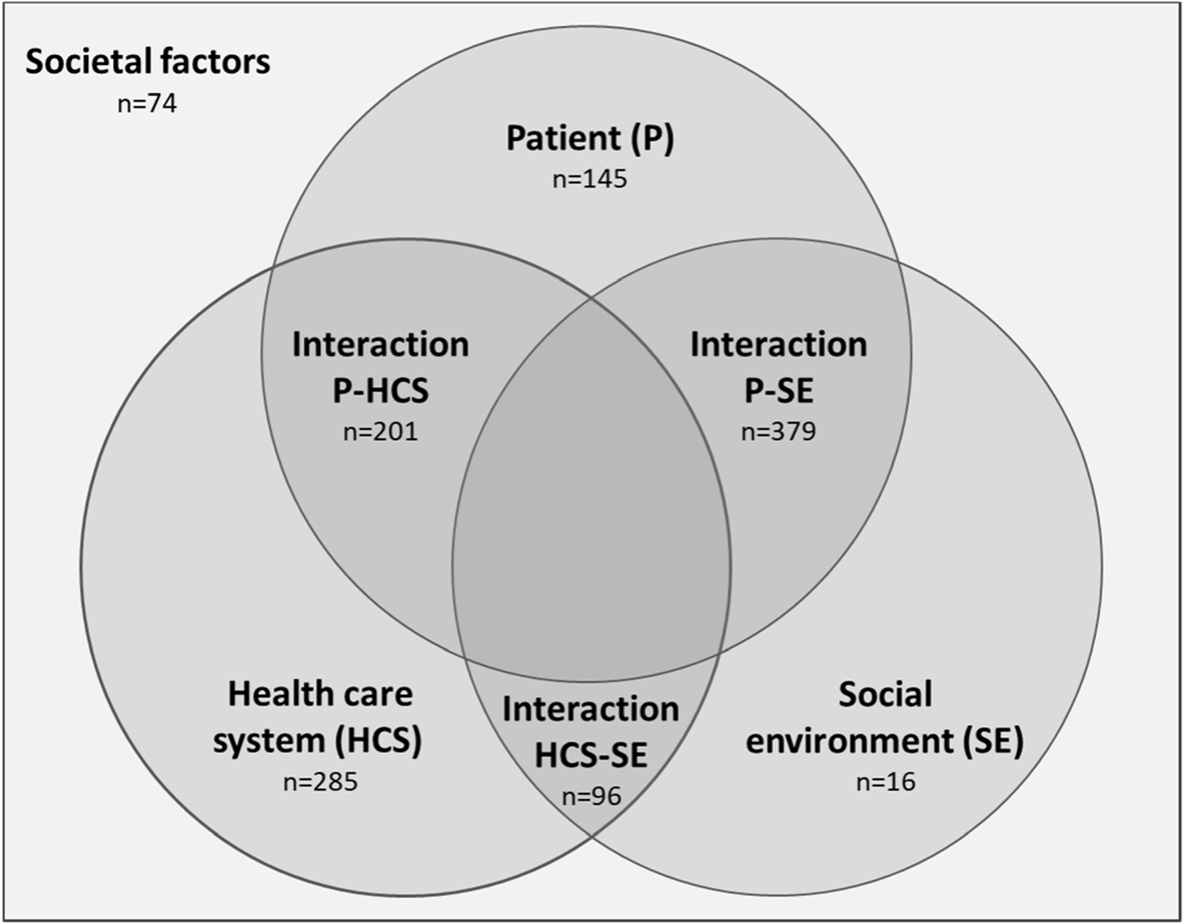
Framework to group facilitators and barriers including the respective number of codings from 22 qualitative interviews

In total, 1.200 codings were assigned. Nearly two third (*n* = 772, 63.28%) of the codings represented a facilitator, and slightly more than one third (*n* = 448, 36.72%) a barrier. The following framework to group facilitators and barriers into broader main categories emerged (s. Figure 1): ‘patient’ (P), ‘social environment’ (SE), ‘health care system’ (HCS) and ‘societal factors’ (SF). Furthermore, the interactions between these categories had to be differentiated. Four codings could not be mapped and belong to a remaining category.

The next level of the developed code tree gives a more detailed description of the facilitators and barriers. Table 1 lists the 20 most frequently mentioned factors (mean rank based on the number of interview partners mentioning the factor and the number of single codings belonging to the factor). Table S1 gives the top 20 list separated by perspectives and age groups (adolescent vs. adult patients). Accordingly, interview partners generally emphasized factors related to their perspective (e.g., professionals mentioning factors within the ‘health care system’). More minor factors are presented in Table S2.

**Table 1:** Top 20 facilitators and barriers in AN treatment initiation

Factor	Main category	Rank^a^	Interview partners n (%)	Single codings n (%)
F + B - (Not) recognizing and addressing	SE-P	1	18 (81.8)	83 (6.9)
F + B – Waiting time and availability	HCS	2	16 (72.7)	82 (6.8)
F+ B – (No/wrong) recommendation and referral	HCS-P	3	20 (90.9)	54 (4.5)
F + B – Competence, specialization, training	HCS	3	13 (59.1)	97 (8.1)
B – Non-understanding of AN as illness or the need for treatment	SE-P	5	12 (54.5)	57 (4.8)
F – Health education and de-stigmatization of AN and psychotherapy	SF	6	14 (63.6)	42 (3.5)
F – Exchange, support, concern, understanding	SE-P	6	13 (59.1)	51 (4.3)
F – Positive role models for treatment	SE-P	8	9 (40.9)	57 (4.8)
F+ B – (No) reminding of, making of or accompanying to appointments	SE-P	9	10 (45.5)	40 (3.3)
F – Suggesting or encouraging to seek treatment	SE-P	10	13 (59.1)	24 (2.0)
F – Somatic symptoms	P	10	10 (45.5)	37 (3.1)
F – Good personal connections	SE-HCS	12	11 (50.0)	33 (2.8)
F + B – Networking, cooperation	HCS	13	7 (31.8)	47 (3.9)
F+ B – (No/vaguely) diagnosing or communicating diagnosis	HCS-P	14	8 (36.4)	34 (2.8)
F+ B – (Not) living, being, eating alone	P-SE	15	10 (45.5)	20 (1.7)
F – Exacerbation and personal breaking point reached	P	16	9 (40.9)	20 (1.7)
B – Trivializing and neglected assistance	HCS-–P	17	^b^6 (27.3)	34 (2.8)
F + B – Fit between individual patient and service settings	HCS	18	7 (31.8)	^b^16 (1.3)
F + B – (Reducing) comparisons with media ideals	SF	19	^b^6 (27.3)	30 (2.5)
F – Continuity (of care) and regular control examinations	HCS-P	20	^b^6 (27.3)	20 (1.7)
**Total**			**22**	**1.200**

*Note*. *F* facilitator, *B* barrier, *SE* social environment, *HCS* health care system, *P* patient, *SF* societal factors, *AN* anorexia nervosa, ^a^ Mean rank based on the rank on the level of interview partners (number of interview partners mentioning the factor) and on the level of codings (number of single text passages mentioning the factor). ^b^ Not among the top 20 within the ranking on the level of interview partners or the level of codings, respectively

### The patient

The most frequently mentioned factors on the part of the patient were ‘somatic symptoms’ (F) and the occurrence of a (symptom) ‘exacerbation or breaking point’ (F). As a reason for seeking help, both adolescent and adult patients mentioned a wide variety of somatic symptoms reaching from brittle nails to pericardial effusion. Regarding the facilitator ‘exacerbation and breaking point’, interview partners stated for example that they were only ready to start treatment when the “limit was reached” (C-771), “it was already so acute” (P-142), they collapsed or had “no real life anymore” (P-765).

### Interactions between the patient and the social environment

In general, most of the statements of our interview partners concerned ‘interactions between the patient and the social environment’ (s. Table 2 for illustrative quotes). First, ‘recognizing and addressing’ (F + B) the changing or worrying eating behaviors, body weight, or physical activities by close others was experienced as important. In some cases, the early detection and confrontation was found to be essential, in other cases continuity and persistence in making AN-symptoms subject of discussion emerged as crucial. A barrier for treatment initiation, which seemed especially relevant to adolescent patients (s. Tab. S1), was the ‘non-understanding of AN as illness and/or of the need for treatment’ (B) within the social environment. Besides an underestimation of the seriousness at early illness stages or in general (e.g., “she will deal with that on her own”) and simplistic or stigmatizing beliefs (e.g., “just eat normal again”), interview partners also pointed to eating disorders of close others as one reason for non-understanding reactions (e.g., formerly anorectic grandmother who strongly supported the thin body ideal). In contrast, the facilitating influence of positive relationships involving ‘exchange, support, concern and understanding’ (F) was mentioned. Support without pressure, open, understanding and calm conversations and expressions of concern were perceived as helpful. In addition to such general social support, interactions with other persons, who already found their way into treatment and experienced improvement or even recovery, were described as a specific facilitative influence on so far untreated persons. Such ‘positive role models for treatment’ (F) were found within the personal lives (e.g., stepsister, colleague) or via (social) media (e.g., Instagram, television documentary). Interview partners stated that positive role models could motivate treatment initiation, answer specific questions about treatments and take fears about treatment. Further facilitative influences relevant for adolescents and adults were seen in practical support by ‘reminding of, making of or accompanying to appointments’ (F + B) and in ‘suggestions and encouragement to seek treatment’ (F). The latter meant for some patients to get an highly important initial external impulse, for others hearing pleas or continuous persuasive efforts, and, usually later in the process, encouraging and motivating words (e.g., “Only way that it will get better.”, P-765). Finally, participants noted the relevance of social resources in general or nearby (‘(Not) living, eating or being alone’ – F + B).

**Table 2:** Illustrating quotes for facilitators and barriers at the interaction of individuals with AN and their social environment

F + B	(Not) recognizing and addressing	“I think it really helped me that somebody saw it and then said to me that this is really a disorder.” (P-709) “My friends reflected on these things and said, “[Patients name], this is not normal!”. Then my mind had to admit that this could be true. I think about it. Reluctantly, but I will.” (P-249)
**B**	**Non-understanding of AN as an illness or of the need for treatment**	“It was that they all [patient’s family] thought I had chosen this disorder […] and I can turn it off and then it’s gone. But it does not work that way. It came gradually and it does not go away simply like pushing a button. And they did not understood this.” (P-765)
**F**	**Exchange, support, concern, understanding**	“And I talked a lot about eating, about these issues with my mother and […] I could basically tell her everything about that and also my worries and my concerns. And I do think that this was not so easy for my mother to hear all this […]. But at least she tried to stay calm and to deal with that in a good way […]. This is what helped me.”(P-989)
**F**	**Positive role models for treatment**	*Interviewer*: “Well, my very last question. If you look back on the whole interview and on all the topics we touched, what would you say, makes it easier for people with anorexia to start treatment?” *Patient*: “I think, well, I would say talking to others and I would say talking a lot to other persons, who went already through this process, I believe. Because they […] can encourage you and they can tell, that it wasn’t that bad, or well how they experienced it.” (P-771)
**F + B**	**(No) reminding of, making of or accompanying to appointments**	“… and if you don’t have this sturdiness. This never ending “I will call again” […]. They were already annoyed with me, but it was useful. She then got the place from 1 day to the other.” (C-194) “For sure, I would say if it were not for my family, I would not have been there. I would not have managed it to do the steps on my own. My husband organized everything. It was clear to me, that I needed it, but … calling, driving there, I would not have done that.” (P-966)
**F**	**Suggesting or encouraging to seek treatment**	“We just told her, that she really needs help and professional help, because we are stuck.” (C-142) „ … I have also motivated her that she keeps going through with her idea […] to go […] in this clinic. And I really motivated her to actually do that, because I thought it was so right.” (C-194)
**F + B**	**(Not) living, being, eating alone**	“… basically only if you are all the time together with her, you could see that it is really nothing she is eating.” (C-249)

*Note*. *F* facilitator, *B* barrier, *P* patient, *AN* anorexia nervosa, *C* carer

### The health care system

One of the most prominent causes for treatment delays were considered to be long ‘waiting times and limited availability’ (F + B, e.g., “I have said, she is starving to death. You can’t tell me [to wait] three months.”, C-194). The interview partners, especially carers, called for more treatment places and better availability of outpatient psychotherapists in general and in rural areas in particular. An important concern was that the tediously build treatment motivation decreases during waiting times. To better address this problem, it was recommended that referring professionals should point out the potentially long waiting times as early as possible. Furthermore, patients often stated to choose the service with the shortest waiting time.

Different statements were concerned with ‘the competence, the specialization and the training’ (F + B) of the referring professionals.. Even though the main tasks of the professionals were merely detection and referral, solid knowledge of the disorder, somatic consequences, and necessary diagnostic procedures (e.g. laboratory parameters), as well as soft skills such as the ability to establish a good patient-physician relationship or communication skills were considered essential. Finding a professional who is competent in dealing with somatic and mental problems was often experienced as a challenge. Two specific suggestions for improvement were mentioned: establishment of a psychosomatic general practitioner and expansion of low-threshold outpatient services located at specialized inpatient facilities. The latter were seen as the best way to assess the need for treatment in a timely manner and to make direct referrals if needed.

Another key factor highlighted by professionals was found to be ‘networking and cooperation’ (F + B). This primarily concerned collaboration between general practitioners and psychotherapists, and secondarily other professions like gynecologists, school psychologists, counselors, and psychiatrists. Physicians experienced the lack of financial compensation for networking and interdisciplinary collaboration to be a hindrance.

The factor ‘fit between patient and service settings’ (F + B) subsumes various, mostly non-modifiable circumstances. Positive influences were, for example, if the clinic was close to home or if fellow patients were expected to be in the same age group. Strongly limited phone times of psychotherapists or time slots for appointments during school or working hours constituted a barrier.

### Interactions between the patient and the health care system

The most important and obvious factor regarding ‘interactions between patient and health care system’ was whether and when the consulted professional referred into specialized treatment (‘(No/wrong) recommendation and referral’ – F + B). Referrals or recommendations which were binding (e.g., “Not only saying ‘think about it’.”, P-966), timely (e.g., with “notification of urgency”, P-249, avoiding prolonged treatments in non-specialized contexts, Pr-709), clear (e.g., “you should, not you could”, C-194) and concrete (i.e., specific provider, C-709, C/Pr-547) were described as facilitative. Additionally, a joint decision making involving carers was described as helpful (Pr-771, C-771).

Prior to the referral, the detection of the disorder and the communication about the diagnosis was indicated to be crucial (‘(No/vaguely) diagnosing or communicating diagnosis’ – F + B). The interview partners described that persons with AN sometimes consult physicians for related reasons or single symptoms (e.g., check for nutritional deficiency or food intolerance, Pr-249; amenorrhea, other mental symptoms, Pr-709). According to various interview partners, especially adult patients, professionals frequently failed to recognize AN in such situations, and an isolated treatment of single symptoms or mental comorbidities was described to prolong treatment initiation. When communicating the diagnosis, patients and carers wished for an unambiguous and clear position of the professional. One adult patient stated receiving her diagnosis in a written form was highly helpful (“copy of the physician’s letter”, P-966). Another option mentioned was to go through each single diagnostic criterion (Pr-709).

A previous experience of ‘trivializing or neglected assistance’ (B) emerged as a clear barrier to treatment. Some patients described that professionals normalized the pathology of the eating disorder (e.g., “And then we went to a general practitioner, but she didn‘t take me seriously. She said, well, that it is normal for adolescents, that it is just a phase in my life, and that we will manage it somehow on our own.”, P-989). Others reported situations in which professionals failed to provide adequate care despite the fact some were severely underweight (e.g., sick leave without follow-up appointment, several emergency room admissions without a correct diagnosis or referral, P-966). After such experiences, it often took time for the individuals to seek help again.

In contrast, ‘continuity (of treatment) and regular control examinations’ (F) performed by the general practitioners were perceived to facilitate a subsequent psychotherapeutic treatment.

### Interactions between social environment and health care system

Sometimes, treatment initiation was also influenced by interactions between carers and professionals. Specifically, existing ‘good personal connections’ (F) between a carer and a professional proved beneficial (e.g., “My grandmother knows this general practitioner and it just so happens the general practitioner is specialized in this field [eating disorders].”, P-142).

### Societal factors

Societal factors affected the female patients, their social environment and professionals. ‘Health education and destigmatization of AN and psychotherapy’ (F) was named as a major societal factor. The interview partners were most likely to associate health education with school, but also mentioned fitness studios. Health education should inform and educate about eating disorders in general and the somatic consequences, the importance of early treatment, and the treatment options in particular. According to the interview partners, it should convey a feeling that there is no need to feel guilt or shame because of the disorder or because of seeking treatment (P-989). The interview partners particularly wished for greater awareness among key persons (e.g., pediatricians, general practitioners, teachers, fitness trainers).

A relevant barrier to treatment was ‘comparisons with media ideals’ (F + B, e.g., “I have to say social media is a big factor [ …] where you are influenced by the body sizes of models and then you think, maybe I look similar to them now, but then why should I be sick?”, P-142). In this context, participants mentioned the removal of pro-Ana content as an important measure.

## Discussion

The main objective of the present study was to identify facilitators and barriers to an early AN treatment using a multi-informant approach involving female adolescent and adult patients, their carers, and referring healthcare professionals. In comparison, facilitating influences were mentioned more frequently than hindering influences. The various interview partners (female patients, carers, professionals) generally focused on facilitators and barriers related to their perspective.

‘Positive role models for treatment’ was a facilitator that specifically stood out in comparison to the existing evidence base. The factor describes a specific positive influence of successfully treated individuals on so far untreated individuals with AN. To our knowledge, this factor has not been mentioned in any previous study. In line with this finding, it was pointed out that there is little research on the sharing of lived experience despite its relevance in the consumer community [33]. In general, positive and negative effects of these ‘interventions’ are to be expected [34, 35], but research has long focused almost exclusively on the negative consequences associated with pro-Ana websites [34–36]. An increase in eating disorder pathology from the use of such websites has been demonstrated [34]. However, it has also been shown that critical and recovery-oriented comments (‘anti-Ana’, ‘pro-recovery’) clearly dominate on social media platforms [37]. Their effects in terms of earlier treatment initiation have not been studied, but the potential active ingredients of recovery-oriented narratives reported in the literature overlap strongly with the statements of our interview partners. These include increases in treatment motivation, hope, knowledge (insider information), and decreases in fear and stigma [33, 38, 39]. Potential risks could include overestimation of treatment effects [39] and maladaptive social comparisons (peer contagion effect) [40, 41]. Nonetheless, based on the present results, it seems highly promising to further investigate the influences of successfully treated former patients (‘positive role models for treatment’) on the treatment initiation of so far untreated individuals with AN.

In addition to the specific facilitative social influence of ‘positive role models’, interactions with family and friends play a crucial role, especially during the period without any contact with health care. The present and previous studies have shown consistently that close others fulfill tasks similar to those of professionals in early stages of illness (e.g. recognition, motivation) and that their recognition (F + B - ‘(not) recognizing and addressing”) [42], understanding (B - ‘non-understanding of AN as illness and/or of the need for treatment’) [19, 43], emotional and practical support (F - ‘exchange, support, concern, understanding’, F + B - ‘(no) reminding of, making of or accompanying to appointments’) [20], and encouragement to seek treatment (F - ‘suggesting or encouraging to seek treatment’) [19, 20], or the lack thereof, may be decisive for treatment initiation. Social problems and isolation (F + B - ‘(not) living, eating, being alone’) can prolong the DUAN [42]. However, the role of close others is also highly challenging. Very little is known about the needs and burden of close others (e.g., need for information, emotional stress, financial strain, impaired social life) specifically in the period before AN treatment starts. It has been suggested that the needs and burden of carers vary depending on the stage of illness [44], but most studies investigated the issue only among carers of AN patients in treatment (e.g. [44, 45]). In early stages, it is essential to recognize the illness, which is often described as a process of gradual realization [46]. According to the present results, adolescents may particularly benefit if carers perceive the difficulties soon in terms of an illness and subsequently recognize the need for professional help (B - ‘non-understanding of AN as illness and/or of the need for treatment’). To achieve such understanding, carers need sound information about AN [46]. Counseling services and support programs for carers can provide such information. Additionally, with regard to their own negative emotions (e.g. guilt, helplessness), maladaptive beliefs and behaviors (e.g. over-involvement, criticism) and possible eating problems of their own, they can provide support and counseling to achieve a better coping with the illness [47]. It seems important that such services are open to close others, even if the person with AN is not yet in treatment. Such an early support of carers may indirectly exhibit a positive influence on the treatment initiation of the individual with AN.

However, considerable delays in treatment also occur within the health care system [13, 17, 18]. In particular, we were able to confirm several findings on the relevance of long waiting times or limited availability (F + B – ‘waiting time and availability’) [13, 19, 20, 48, 49] and past negative experiences with health care professionals (B – ‘trivializing and neglected assistance’) [19, 20, 43, 48, 49]. Both factors strongly indicate weaknesses within the health care system. Especially, the trivialization of symptoms by professionals obviously counteracted early diagnosis and treatment. Thereby, professionals seem to reinforce maladaptive thoughts of individuals with AN that the problem might be “not serious enough” [48] or that there must be a symptom exacerbation (F – ‘exacerbation or breaking point’) [50] and/or (severe) ‘somatic symptoms’ (F) [19, 50] before treatment is initiated. In other instances, and more frequently, professionals failed to make a diagnosis or refer to specialized treatment (F + B – ‘(no/wrong) recommendation and referral’, F + B – ‘(no/vague) diagnosing or communicating diagnosis’) [49, 51]. Thiswas often attributed to a lack of ‘competence, specialization, or training’ (F + B) regarding eating disorders, especially in primary care [49]. In addition to more intensive training, screening and decision support tools are often recommended [49, 52]. However, the presently interviewed professionals also emphasized the importance of ‘networking and collaboration’ (F + B) at the interface between primary and secondary healthcare.

Finally, the improvement of mental health literacy [19, 48] can be an important societal factor. Health education and awareness programs (F – ‘health education and de-stigmatization of AN and psychotherapy’) can reduce stigma and negative attitudes related to the disorder and the treatment, which are often cited as a barrier to early treatment [19, 20, 43, 48, 50].

### Strengths and limitations

The present findings have to be viewed in light of the strengths and weaknesses of our study. To our knowledge, this is the first study to investigate facilitators and barriers to AN treatment initiation using a multi-informant approach. Therefore, we could shed light on factors relevant to female adolescent and adult patients, carers and professionals. Prior studies often focused solely on the perspective of adult patients. It can be argued that the present sample size within each group (e.g., adolescent patients) is relatively small. However, the overall sample size of our study is well within the recommended range [28]. Furthermore, the correct and careful selection of study participants in order to reach theoretical and data saturation is often higher valued in qualitative research than the mere number of study participants [28, 53]. Accordingly, we applied rigorous eligibility criteria (e.g., minimum age, beginning of the first treatment no longer than 3 months ago) and followed a pre-defined sampling scheme. As one consequence of this sampling scheme, we could achieve a wide range of the DUAN within our study sample. However, regarding our sampling, it is also important to note that male patients as well as individuals who never entered specialized treatment were not included in the present analysis. Additionally, two of the general practitioners interviewed had a specialty in eating disorders, which seems atypical. In general, we could gain insights on the spectrum of factors relevant to AN treatment initiation, but we cannot make conclusions about the relative importance of certain facilitators or barriers. Additional quantitative evidence especially on modifiable facilitators and barriers is needed for this purpose. In order to subsequently develop an instrument for the assessment of facilitators and barriers and to gain such evidence (FABIANA sub-studies II and III), the present analyses remained relatively specific and concrete. Consequently, we did not consider potential meta-themes within the qualitative material.

## Conclusion

In conclusion, our study set out to better understand the spectrum of facilitators and barriers in AN treatment initiation using a multi-informant approach. Most of the presently identified facilitators and barriers seem to hold potential for modifiability and improvements. Our findings suggest that measures to decrease the duration of untreated AN and avoid chronic illness courses might not only address patients and the health care system, but may also involve close others and successfully treated former patients. A newly identified specific measure to improve the treatment initiation of untreated individuals with AN might be the use of treatment narratives from successfully treated former patients.

## Supplementary Information

**Additional file 1.**

**Additional file 2.**

## Data Availability

The anonymized interview transcripts will be made available by the authors upon legitimate request.

## Abbreviations

AN: Anorexia nervosa
B: Barrier
BMI: Body-Mass-Index
C: Carer or close other
DSM-IV: Diagnostic and Statistical Manual of Mental Disorders – Fourth Edition
DUAN: Duration of untreated Anorexia nervosa
F: Facilitator
HCS: Health care system
P: Patient
Pr: Health care professional
SCID I/II: Structured clinical interview for DSM-IV Axis I/II diagnoses
SE: Social environment
SF: Societal factors
