# Supplementary material for: Facilitators and barriers in anorexia nervosa treatment initiation: a qualitative study on the perspectives of patients, carers and professionals
Source: J Eat Disord. 2021 Feb 27;9:28. doi: 10.1186/s40337-021-00381-0 (PMC7913310; doi:10.1186/s40337-021-00381-0)
Supplement: Supplementary file 1 — Additional file 1. [file 40337_2021_381_MOESM1_ESM.docx]

| **Factor** | **Topic** | **Rank^†^** | **Level of interview partner** | | | | | | **Level of Codings** | | | | | |
| --- | --- | --- | --- | --- | --- | --- | --- | --- | --- | --- | --- | --- | --- | --- |
|  |  |  | Adol | Adult | Carer | Profess | C-P | Total | Adol | Adult | Carer | Profess | C-P | Total |
| F+B - (Not) recognizing and addressing *n (%)* | SE(-P) | 1 | 3 (75) | 6 (100) | 6 (85.7) | 2 (50) | 1 (100) | 18 (81.8) | 13 (6.2) | 33 (12.3) | 31 (7.8) | 2 (0.8) | 4 (6.7) | 83 (6.9) |
| F+B – Waiting time and availability *n (%)* | HCS | 2 | 3 (75) | 3 (50) | 5 (71.4) | 4 (100) | 1 (100) | 16 (72.7) | 4 (1.9) | 8 (3) | 49 (12.3) | 13 (5) | 8 (13.3) | 82 (6.8) |
| F+ B - (No/wrong) recommendation and referral *n (%)* | HCS (-P) | 3 | 3 (75) | 6 (100) | 6 (85.7) | 4 (100) | 1 (100) | 20 (90.9) | 5 (2.4) | 11 (4.1) | 19 (4.8) | 17 (6.5) | 2 (3.3) | 54 (4.5) |
| F+B – Competence. specialization. training *n (%)* | HCS | 3 | 1 (25) | 3 (50) | 4 (57.1) | 4 (100) | 1 (100) | 13 (59.1) | 1 (0.5) | 7 (2.6) | 15 (3.8) | 70 (26.7) | 4 (6.7) | 97 (8.1) |
| B – Non-understanding illness or need for treatment *n (%)* | SE(-P) | 5 | 3 (75) | 2 (33.3) | 4 (57.1) | 2 (50) | 1 (100) | 12 (54.5) | 18 (8.5) | 5 (1.9) | 21 (5.3) | 9 (3.4) | 4 (6.7) | 57 (4.8) |
| F – Health education & de-stigmatization of AN and psychotherapy *n (%)* | SF | 6 | 3 (75) | 4 (66.7) | 4 (57.1) | 2 (50) | 1 (100) | 14 (63.6) | 8 (3.8) | 12 (4.5) | 13 (3.3) | 8 (3.1) | 1 (1.7) | 42 (3.5) |
| F – Exchange. support. concern. understanding *n (%)* | SE (-P) | 6 | 3 (75) | 3 (50) | 6 (85.7) | 1 (25) | 0 | 13 (59.1) | 22 (10.4) | 11 (4.1) | 17 (4.3) | 1 (0.4) | 0 (0) | 51 (4.3) |
| F – Positive role models for treatment *n (%)* | SE (-P) | 8 | 2 (50) | 2 (33.3) | 5 (71.4) | 0 | 0 | 9 (40.9) | 18 (8.5) | 8 (3) | 31 (7.8) | 0 | 0 | 57 (4.8) |
| F+ B - (No) reminding of. making of or accompanying to appointments *n (%)* | SE (-P) | 9 | 2 (50) | 3 (50) | 4 (57.1) | 0 | 1 (100) | 10 (45.5) | 9 (4.3) | 19 (7.1) | 10 (2.5) | 0 | 2 (3.3) | 40 (3.3) |
| F – Suggesting or encouraging to seek treatment *n (%)* | SE (-P) | 10 | 3 (75) | 4 (66.7) | 5 (71.4) | 1 (25) | 0 | 13 (59.1) | 6 (2.8) | 6 (2.2) | 11 (2.8) | 1 (0.4) | 0 | 24 (2) |
| F – Immanent to AN – somatic symptoms *n (%)* | P | 10 | 2 (50) | 4 (66.7) | 2 (28.6) | 2 (50) | 0 | 10 (45.5) | 12 (5.7) | 18 (6.7) | 5 (1.3) | 2 (0.8) | 0 | 37 (3.1) |
| F – Good connections *n (%)* | SE-HCS | 12 | 2 (50) | 2 (33.3) | 3 (42.9) | 3 (75) | 1 (100) | 11 (50) | 2 (0.9) | 5 (1.9) | 14 (3.5) | 9 (3.4) | 3 (5) | 33 (2.8) |
| F+B – Networking. cooperation *n (%)* | HCS | 13 | 1 (25) | 1 (16.7) | 2 (28.6) | 3 (75) | 0 | 7 (31.8) | 1 (0.5) | 2 (0.7) | 3 (0.8) | 41 (15.6) | 0 | 47 (3.9) |
| F+ B - (No/vaguely) diagnosing or communicating diagnosis *n (%)* | HCS (-P) | 14 | 0 | 3 (50) | 2 (28.6) | 3 (75) | 0 | 8 (36.4) | 0 | 18 (6.7) | 8 (2) | 8 (3.1) | 0 | 34 (2.8) |
| F+ B - (Not) living. being. eating alone *n (%)* | P (-SE) | 15 | 2 (50) | 3 (50) | 2 (28.6) | 2 (50) | 1 (100) | 10 (45.5) | 4 (1.9) | 4 (1.5) | 7 (1.8) | 4 (1.5) | 1 (1.7) | 20 (1.7) |
| F - Immanent to AN – exacerbation and personal breaking point reached *n (%)* | P | 16 | 2 (50) | 3 (50) | 3 (42.9) | 1 (25) | 0 | 9 (40.9) | 3 (1.4) | 6 (2.2) | 10 (2.5) | 1 (0.4) | 0 | 20 (1.7) |
| B – Trivializing & neglected assistance* *n (%)* | HCS (-P) | 17 | 2 (50) | 1 (16.7) | 0 | 2 (50) | 1 (100) | 6 (27.3) | 7 (3.3) | 7 (2.6) | 0 | 5 (1.9) | 15 (25) | 34 (2.8) |
| F+B – Fit between individual patient and service settings* *n (%)* | HCS | 18 | 2 (50) | 1 (16.7) | 4 (57.1) | 0 | 0 | 7 (31.8) | 2 (0.9) | 2 (0.7) | 12 (3) | 0 | 0 | 16 (1.3) |
| F + B – (Reducing) comparisons with media ideals* *n (%)* | SF | 19 | 1 (25) | 2 (33.3) | 2 (28.6) | 1 (25) | 0 | 6 (27.3) | 2 (0.9) | 9 (3.3) | 18 (4.5) | 1 (0.4) | 0 | 30 (2.5) |
| F – Continuity (of treatment) and regular control examinations* *n (%)* | HCS (-P) | 20 | 2 (50) | 1 (16.7) | 2 (28.6) | 1 (25) | 0 | 6 (27.3) | 11 (5.2) | 3 (1.1) | 2 (0.5) | 4 (1.5) | 0 | 20 (1.7) |
| **Total** |  |  | 4 | 6 | 7 | 4 | 1 | **22** | 211 | 269 | 398 | 262 | 60 | **1.200** |

**Tab. S1.** Top 20 facilitators and barriers of AN treatment initiation separated by perspectives

E – social environment, HCS – health care system, P – patient, SF – Societal factors, AN: Anorexia nervosa, Adol: adolescents, C-P: Carer and professional, Profess: professional, † Mean rank based on the rank on the level of interview partners and on

the level of codings. * Not among the Top 20 within either the separate ranking of the level of interview partners or the level of codings.
