# Supplementary material for: Facilitators and barriers in anorexia nervosa treatment initiation: a qualitative study on the perspectives of patients, carers and professionals
Source: J Eat Disord. 2021 Feb 27;9:28. doi: 10.1186/s40337-021-00381-0 (PMC7913310; doi:10.1186/s40337-021-00381-0)
Supplement: Supplementary file 2 — Additional file 2. [file 40337_2021_381_MOESM2_ESM.docx]

| **Main category** | **Factor** | **Example** |
| --- | --- | --- |
| P | B - ‘Shame, stigma and fearing the reactions of others’ | “Sure, everyone saw it, but I did not want to make it really official…that it gets out this way”, (P-966) |
| P | B - ‘Concerns regarding treatment-caused absence in school, work or childcare’ | “Often young women with children say ‘I cannot be away from home’. It is not possible.” (Pr-771) |
| P | F - ‘Conflict between AN and other motives’ | “I always had big wishes and plans and dreams and I realized that this is not working anymore”, (P-194) |
| P | F - ‘Role as a mother’ | “But I am a mother, who cannot just crumble away in front of her child. That’s when I realized, that there is something not healthy.”, (P-249) |
| SE | F - ‘Exchange within the social environment’ | “Yes, it was unpleasant that other people said something, but it is important, because you get a little bit blind towards the whole issue.”, (C-793) |
| SE | F - ‘Knowledge about and experiences with eating disorders’ | “The daughter of the new partner of my mother also had AN. She is older, but I know, she was also in treatment. […] I believe this was the reason, why she recognized it relatively early.”, (P-771) |
| HCS | F + B - ‘Information policy and the professional web presence’ | “I checked the website of the clinic and it sounded really good and everything was presented in a way that there is really no problem with seeking help.”, (P-709) |
| P – HCS | F + B - ‘(No) positive patient-physician relationship’ | “She tried to take my fears […] and I simply felt supported and that there is a person who is really interested to help”, (P-771) |
| P – HCS | B - ‘Delays due to necessary referral letters, medical reports or cost agreements’, | “…then the general practitioner wrote a referral letter for the clinic, but the clinic said, no, this cannot be from the general practitioner, we need this from a psychotherapist”, (C-194) |
| P – HCS | F - ‘Setting and communicating of clear limits’ | “They [general practitioners] treat a lot of patients in outpatient care, but they have to define a very, very clear [weight] limit, when they say, now you have to go in a clinic”, (Pr-709) |
| P – HCS | F - ‘Pointing to potential or existing somatic consequences’ | “I had the impression that it was not her own insight […], but rather that all doctors told her that the pericardial effusion is still there and the liver values are increased and thyroid values are bad”, (C-142) |
| SE – HCS | F - ‘Exchanges between the carer and the physician’ | “…and then there was a connection between her [general practitioner] and my parents”, (P-142) |

**Tab. S2** Minor facilitators and barriers of AN treatment initiation sorted by main category

Note. F – facilitator, B – barrier, P – patient , SE – social environment, HCS – health care system, ,AN - anorexia nervosa.
